# Identification of *Codonopsis Radix* from different origins based on odor information

**DOI:** 10.3389/fnut.2025.1536189

**Published:** 2025-02-14

**Authors:** Xingyu Guo, Ruiqi Yang, Jiayu Wang, Yushi Wang, Shulin Yu, Huiqin Zou, Yonghong Yan

**Affiliations:** School of Chinese Materia Medica, Beijing University of Chinese Medicine, Beijing, China

**Keywords:** *Codonopsis Radix*, HPLC fingerprint, E-nose, SPME-GC–MS, geographical origin discrimination

## Abstract

**Introduction:**

*Codonopsis Radix*, a natural plant with edible and medicinal functions, is in high demand and cultivated in different regions. The place of origin affects the quality. However, environmental factors such as soil, climate, and altitude all have an impact on its quality. Therefore, identification of geographical origins of *Codonopsis Radix* is very important.

**Methods:**

This study used High Performance Liquid Chromatography (HPLC) fingerprint and Electronic Nose (E-nose) technology to identify the origin information of *Codonopsis Radix*. HPLC fingerprint can fully reflect characteristics of chemical components in *Codonopsis Radix* and provide a reliable chemical basis for identification of origins, while E-nose technology captures and analyzes volatile odor of *Codonopsis Radix* by simulating human olfactory system, which realizes rapid identification of origin information. In order to reveal origin of special aroma of *Codonopsis Radix* in a deeper way, we further employed Solid Phase Micro-extraction Gas Chromatography-mass Spectrometry (SPME-GC-MS) technique to elucidate the volatile substances of unique aroma.

**Results:**

Odor information could replace chemical components to achieve the identification of *Codonopsis Radix* geographical origins and Hexanal might be a key volatile compound in *Codonopsis Radix* from different geographical origins.

**Discussion:**

This research indicated that *Codonopsis Radix* from different origins can be identified by odor information, which not only enriched index system for quality evaluation of *Codonopsis Radix*, but also provided new ideas and methods for origin identification of same type of plants.

## 1 Introduction

*Codonopsis Radix* is a tonic traditional Chinese medicine, first used as a substitute for *Ginseng Radix et Rhizoma*, with anti-inflammatory, antioxidant, endocrine regulation, anti-aging, and other effects (1). In 2018, the National Health Planning Commission approved *Codonopsis Radix* as a variety of homology of medicine and food (2). *Codonopsis Radix* is native to the Shangdang area of Shanxi Province. Due to the increasing scarcity of wild resources, cultivated products are now the mainstream in the market. According to reports, annual demand for *Codonopsis Radix* can reach 35,000 tons, of which 25% is used for food and 11% for export. As the market continues to expand, more and more regions have begun to cultivate it. However, studies have shown that the origins of *Codonopsis Radix* have different qualities (3), and the reason may be related to ecological factors of the cultivation environment (4). Therefore, it is necessary to identify the geographical origins of *Codonopsis Radix* accurately.

Currently, common origin identification techniques include spectroscopy (5), chromatographic technology (6), and molecular biology technology (7). All of them are accurate and reliable. Moreover, *Codonopsis Radix* has a special aroma. With this in mind, can we use aroma to identify the geographical origin information of *Codonopsis Radix*? E-nose is a biomimetic technology with quick and accurate systems for recognizing odors (8). More importantly, it can reduce the use of organic reagents, which is helpful for environmental protection. Hence, E-nose is gradually being introduced into food and pharmaceutical sectors. For example, Zhang J.B. et al. used Flash GC E-nose and Headspace Gas Chromatography–Mass Spectrometry to recognize *Ziziphi Spinosae Semen* of different geographical origins (9).

In this study, we collected samples of *Codonopsis Radix* from six mainstream producing areas. First, HPLC was used for sample classification as a frame of reference for follow-up studies. Second, E-nose was used to obtain the odor profiles of the samples. Third, SPME-GC–MS was used to elucidate volatile substances of *Codonopsis Radix*. Finally, we used various machine learning to identify different origins. This study can provide a reference for the identification of other products of the same type.

## 2 Materials and methods

### 2.1 Samples, instruments, and reagents

#### 2.1.1 Samples

Based on literature and market conditions, we chose six main production areas of *Codonopsis Radix*, and a total of 77 batches of samples were collected. To ensure the reliability of sample source information, we chose to purchase the samples directly from farmers or suppliers in origin. Among them, Shanxi-produced *Codonopsis pilosula* (Franch.) Nannf. were purchased from Longxi Town, Pingshun County, Changzhi City; Gansu-produced *Codonopsis pilosula* (Franch.) Nannf. were purchased from Qingshui Town, Min County, Longxi City, and Dacaotan Town, Zhang County; Gansu-produced *Codonopsis pilosula* Nannf. var. *modesta* (Nannf.) L.T.Shen were purchased from Baoziba Town, Wen County, Longnan City; Sichuan-produced *Codonopsis pilosula* Nannf. var. *modesta* (Nannf.) L.T.Shen were purchased from Jiuzhai County, Aba Tibetan and Qiang Autonomous Prefecture; Chongqing-produced *Codonopsis tangshen* Oliv. were purchased from Hongchun Township, Wushan County; and Hubei-produced *Codonopsis tangshen* Oliv. were purchased from Banqiao Town, Enshi City. The collection time was all in the autumn and winter of 2023. Sample information is shown in Table 1.

**Table 1:** Information of samples.

Variety	Origin	Batch	Number
*Codonopsis pilosula* (Franch.) Nannf.	Shanxi	14	DS-SX-1 ~ DS-SX-14
	Gansu	13	DS-GS-1 ~ DS-GS-13
*Codonopsis pilosula* Nannf. var. *modesta* (Nannf.) L.T.Shen	Gansu	8	SH-GS-1 ~ SH-GS-8
	Sichuan	15	SH-SC-1 ~ SH-SC-15
*Codonopsis tangshen* Oliv.	Chongqing	15	CD-CQ-1 ~ CD-CQ-15
	Hubei	12	CD-HB-1 ~ CD-HB-12

#### 2.1.2 Instruments

The following instruments were used for this study: High-speed universal grinding model (Beijing Kewei Yongxing Instrument Co., Ltd., China); Electronic Balance (Chengdu Besec Instrument Research Institute, China); CNC ultrasonic cleaning (Kunshan Ultrasonic Instrument Co., Ltd., China); Waters e2695 High-Performance Liquid Chromatograph (Waters 2,998 DAD Detector, USA); Agilent ZORBAX SB-C18 Column (4.6 × 250 mm, 5 μm, USA); *α*-Fox 4,000 Odor Fingerprint Analyzer (Alpha M.O.S, France); CAR/DVB/PDMS Solid Phase Micromanipulation Fiber (Supelco, USA); 7890B GC-59771MS GC–MS (Agilent, USA); and HP-5MS Ultra-Inert Capillary Column (30 m × 250 μm × 0.25 μm, USA).

#### 2.1.3 Reagents

The following reagents were used for this study: Adenosine Control (Lot Code: CFS202301, Purity≥98.0%), Tryptophan Control (Lot Code: CFS202301, Purity≥98.0%), Syringin Control (Lot Code: CFS202302, Purity≥98.0%), Tangshenoside I Control (Lot Code: CFS202301, Purity≥98.0%), and Lobetyolin Control Substance (Lot Code: CFS202302, Purity≥98.0%). These reference substances are all from Wuhan Tianzhi Biotechnology Co., Ltd. Lobetyolinin (Lot Code: CHB200721, purity ≥98.0%) was purchased from Chengdu Cloma Biotechnology Co., Ltd.

### 2.2 HPLC fingerprint analysis

A test solution was prepared by referring to relevant literature (10). An accurate weight of 2 g of *Codonopsis Radix* powder was taken; an ultrasonic extraction method was used to prepare the test solution. In this, the extraction solvent was 20 mL of 70% methanol, ultrasonic power was 250 W, and extraction time was 30 min. The control solution was prepared in methanol, including adenosine, tryptophan, syringin, tangshenoside I, lobetyolinin, and lobetyolin.

The detection wavelength was 260 nm, the injection volume was 20 μL, the column temperature was 20°C, and the flow rate was 0.8 mL/min. Mobile phase A was acetonitrile, and Mobile phase B was 0.15% formic acid solution. Gradient elution was as follows: 0–15 min, 5–6% (A); 15–20 min, 6–10% (A); 20–37 min, 10–14% (A); 37–42 min, 14–19% (A); 42–66 min, 19–30% (A); 66–69 min, 30–55% (A); 69–76 min, 55–58% (A); 76–79 min, 58–70% (A); 79–84 min, 70–5% (A); 84–90 min, 5% (A). The Relative standard deviation (RSD) value of less than 3% was adopted as the benchmark for methodological assessments, encompassing precision, stability, and reproducibility. Chromatographic Fingerprint Similarity Evaluation System 2012 was used to draw fingerprints.

### 2.3 E-nose analysis

A commercial *α*-Fox 4,000 equipped with 18 metal-oxide semiconductors was used. A batch of *Codonopsis Radix* was taken to determine the detection conditions. Acquisition time was 120 s.

### 2.4 SPME-GC–MS analysis

Samples were weighed and incubated at the same temperature as the E-nose, placed in 20 mL headspace injection bottles, extracted at 40°C for 30 min, inserted the extraction head into the injection port, and desorbed at 250°C for 5 min. The carrier gas was helium, the flow rate was 1 mL/min, full scan mode, the ion source temperature was 230°C, and quadrupole temperature was 150°C. The heating program was as follows: 40°C for 8 min, 8°C/min to 125°C for 10 min, 10°C/min to 150°C for 5 min, and 20°C/min to 250°C for 10 min. For extraction temperature and other experimental parameters, refer to E-nose test conditions and related literature (11). The NIST14 database was used to match the compounds obtained by mass spectrometry.

### 2.5 Data analysis

SIMCA14.1 was used for Orthogonal Partial Least Squares Discriminant Analysis (OPLS-DA) analysis, Weka 3.8.6 was used to select the classification model, and Origin 2021 was used to draw a stacked histogram, box plot, and heat map.

## 3 Results

### 3.1 HPLC fingerprint analysis

#### 3.1.1 Methodological investigation

Precision: *Codonopsis Radix* CD-HB-11 sample was taken to prepare a test solution, and six needles were injected continuously. The highest peak was taken as a reference. The relative retention time RSD of the common peak was below 1.00%, and the relative peak area RSD was below 4.10%, indicating that the precision of the instrument was qualified. Stability: *Codonopsis Radix* CD-HB-11 sample was taken to prepare a test solution, injected at 0, 3, 6, 12, and 24 h. Similarly, the highest peak was taken as a reference. The relative retention time RSD of the common peak was below 2.48%, and the relative peak area RSD was below 4.86%, indicating that the test solution was stable within 24 h. Repeatability: Six test solutions were prepared in parallel from the *Codonopsis Radix* CD-HB-11 sample. The relative retention time RSD of the common peak was below 0.77% and the relative peak area RSD was below 4.72%, indicating that the method had good repeatability.

#### 3.1.2 Similarity analysis

**Figure 1:**
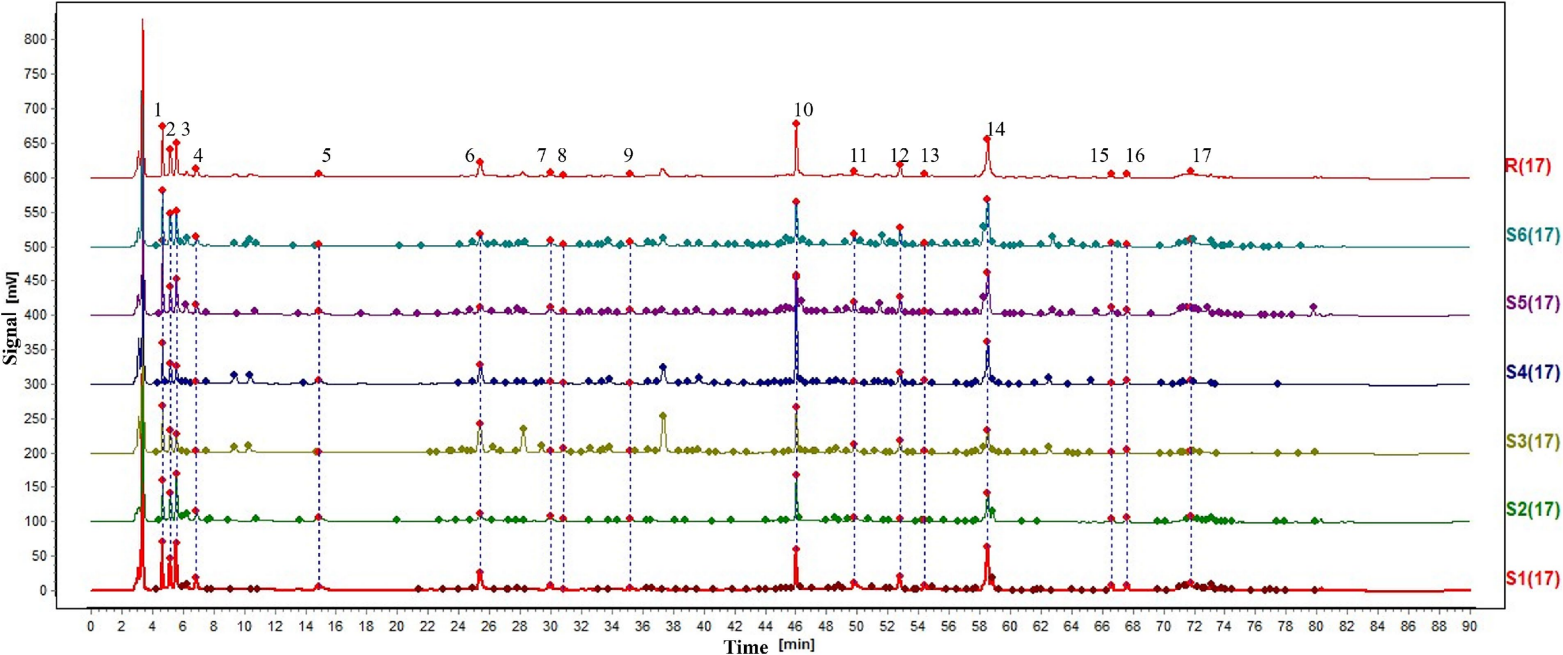
HPLC fingerprint. 3-Adenosine. 6-Tryptophan. 8-Syringin. 10-Tangshenoside I. 12-Lobetyolinin. 14-Lobetyolin.

A total of 77 batches of samples were measured according to the above preparation and detection methods, and the results were saved in cdf. Format and imported into Chromatographic Fingerprint Similarity Evaluation System 2012. Selected samples from different origins were used to obtain the graphs; as shown in Figure 1, 17 common peaks were identified. In the next part, we identified six known peaks by comparing the control solution; peaks 3, 6, 8, 10, 12, and 14, in Figure 1, corresponded to adenosine, tryptophan, syringin, tangshenoside I, lobetyolinin, and lobetyolin. The results showed that the similarity of 77 batches of samples ranged from 0.442 to 0.999, indicating that different samples have different chemical compositions.

#### 3.1.3 OPLS-DA analysis

**Figure 2:**
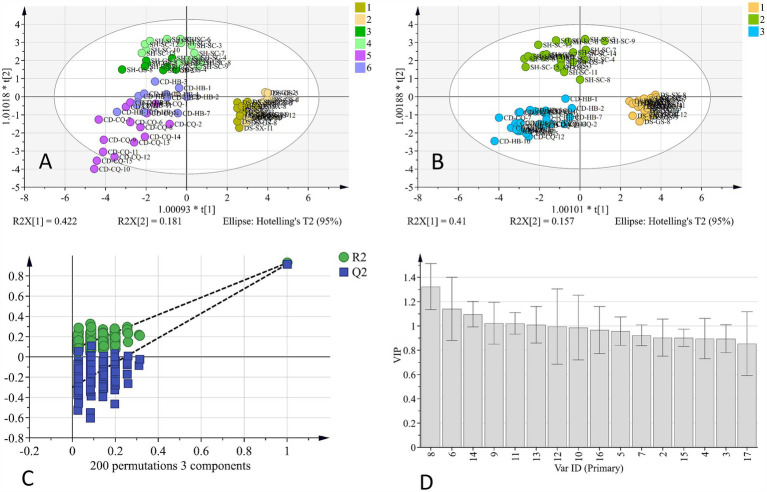
OPLS-DA analysis of HPLC fingerprint. **(A)** OPLS-DA analysis of geographical origins. **(B)** OPLS-DA analysis of varieties. **(C)** 200 permutation test of OPLS-DA. **(D)** VIP value of OPLS-DA.

OPLS-DA is a statistical method for supervised discriminant analysis. By filtering out information, “noise,” that is not related to classification, the constructed model can focus on core classification features and thus has a higher parsing ability and prediction accuracy. OPLS-DA analysis results of the HPLC fingerprint are shown in Figure 2, and *R^2^x* was 0.891, *R^2^y* was 0.725, and *Q^2^* was 0.667. From Figure 2A, it can be seen that the distinction between samples of different origins can be basically realized, but different origins from the same variety were slightly overlapped. This may indicate that variety is the critical factor affecting the quality of *Codonopsis Radix* and that two origins chosen for the same variety were geographically closer and thus more similar in terms of environmental factors, so the difference in chemical composition was not significant. Next, we used OPLS-DA to analyze different varieties of *Codonopsis Radix*; the results are shown in Figure 2B, and *R^2^x* was 0.856, *R^2^y* was 0.907, *Q^2^* was 0.892, all of which were greater than 0.5, indicating that the results had a good reference. In addition, the permutation test showed that the intersection point of the *Q^2^* regression line and longitudinal axis was less than 0 (Figure 2C). Therefore, the model was not overfitted, and the results were valid. Therefore, the results of this analysis had high validity and credibility.

The obtained VIP value of OPLS-DA analysis (Figure 2D), VIP > 1, indicates that the component is statistically significant for distinguishing samples. The results suggest that peaks 8, 6, 14, 9, 11, and 13 VIP values exceeded this threshold, indicating that they were important for distinguishing *Codonopsis Radix* samples, and 8, 6, and 14 were attributed to syringin, tryptophan, and lobetyolin, and the VIP values were 1.324, 1.140, and 1.097, respectively. In this study, the three are often used as indicators to evaluate the quality of *Codonopsis Radix* (12), among which the activity of lobetyolin is more abundant. Lobetyolin has not only antioxidant and anti-inflammatory activities but also shows potential applications in cardiovascular protection, anti-tumor, etc. (13). To sum up, it was reliable to identify the origins of *Codonopsis Radix* by chemical composition. By analyzing chemical composition, especially focusing on key components with high VIP values, such as syringin, tryptophan, and lobetyolin, we were able to reliably differentiate between samples of *Codonopsis Radix* from different origins.

### 3.2 E-nose analysis

#### 3.2.1 Detection parameters

A single-factor investigation method was used to determine the detection parameters based on the maximum response value of the sensor as well as the RSD value of the maximum response value (Table 2). The optimal incubation temperature was 40°C, which is closer to human body temperature and ambient temperature and could reflect the real odor of *Codonopsis Radix* to a certain extent. Therefore, this temperature would also be used as the sample incubation condition for SPME-GC–MS.

**Table 2:** Detection parameters.

Parameter	Condition	Parameter	Condition
Sample size	0.6 g	Injection speed	2000 μL/s
Acquisition duration	120 s	Incubation time	5 min
Acquisition time	600 s	Incubation temperature	40°C
Flow	150 mL/min	Agitation speed	250 rpm
Injection volume	2000 μL	Syringe temperature	50°C

#### 3.2.2 E-nose test results

**Figure 3:**
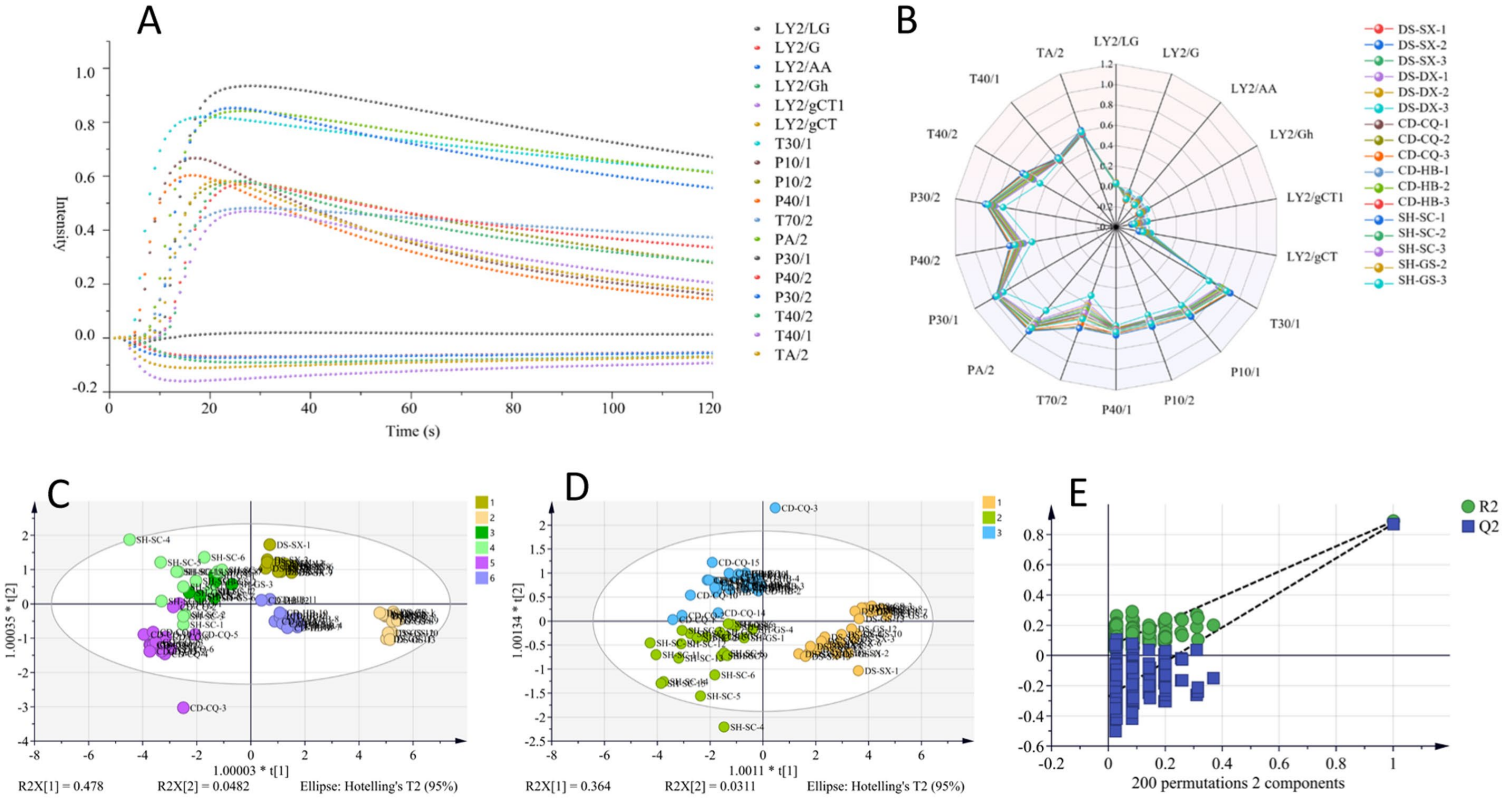
OPLS-DA analysis of E-nose. **(A)** Maximum response of each sensor (DS-SX-1). **(B)** Radar diagram. **(C)** OPLS-DA analysis of geographical origins. **(D)** OPLS-DA analysis of varieties. **(E)** 200 permutation test of OPLS-DA.

In total, 77 batches of samples were measured according to optimal detection parameters, and the maximum response of each sensor was selected as the characteristic value of the samples, taking DS-SX-1 as an example, and response values are shown in Figure 3A. The radar diagram of maximum response value of each sensor for different *Codonopsis Radix* is shown in Figure 3B. From the diagram, it could be seen that response values of sensors TA/2, T40/1, T40/2, P30/2, P40/2, P30/1, PA/2, T70/2, P40/1, P10/2, P10/1, and T30/1 were larger for different *Codonopsis Radix* samples, and changes of T40/2, P30/2, P40/2, PA/2, T70/2, and T30/1 sensors were more obvious. Generally, these six sensors were more sensitive to substances such as ketones, ethanol, ammonia, organic amines, aromatic compounds, organic compounds, and gases with strong oxidizing ability, indicating that there were differences in odors among different *Codonopsis Radix* samples, which might be related to types or contents of above substances.

#### 3.2.3 OPLS-DA analysis

SIMCA 14.1 was used for OPLS-DA analysis, and the results are shown in Figure 3. *R^2^x,* for distinguishing *Codonopsis Radix* from different origins, was 0.999, which indicated that the model had an excellent fit to sample data, *R^2^y* was 0.669, and *Q^2^* was 0.494. These two indicators showed predictive power, but there was still room for improvement. Moreover, it could be seen from Figure 3C that the samples of Gansu-produced and Sichuan-produced, as well as some of the Chongqing-produced and Sichuan-produced, overlapped, and they could not be separated entirely, so it was necessary to explore the other classification models further. In addition, what about the classification of different varieties of *Codonopsis Radix* based on OPLS-DA? The results showed that *R^2^x* was 0.997, *R^2^y* was 0.749, and *Q^2^* was 0.621, and the permutation test was effective (Figures 3D,E). In summary, although the OPLS-DA model had challenges in distinguishing party *Codonopsis Radix* samples from some origins, it showed better results in distinguishing different varieties of *Codonopsis Radix* samples.

#### 3.2.4 Machine learning

Weka 3.8.6 was used to construct classification models carried out by the eigenvalues obtained from E-nose detection, and the accuracy of the classification models was evaluated by 10-fold cross-validation and external tests (Table 3). Among them, Correctly Classified Instances of MultiClassClassifier and Logistic were 100%. Usually, MultiClassClassifier is different from binary classification and can accurately classify multi-class samples. Logistic regression analysis is a mature classification method with the advantages of accuracy and fast training speed. Compared with other classification algorithms, MultiClassClassifier and Logistic could extract effective information from E-nose eigenvalues and realize accurate discrimination of *Codonopsis Radix* from different origins.

**Table 3:** Correctly classified instances of different classifiers.

Classifier	Correctly classified instances (%)	
	10-fold cross-validation	External test (70%)
Multilayer perceptron	94.81	95.65
MultiClassClassifier	100.00	100.00
Simple logistic	92.21	95.65
IBK	94.81	95.65
KStar	93.51	95.65
Logistic	100.00	100.00

### 3.3 SPME-GC–MS analysis

#### 3.3.1 Detection results

GC–MS results of *Codonopsis Radix* powder directly incubated at high temperature were unsatisfactory, whereas the identification of samples by E-nose could be achieved at an incubation temperature of 40°C, which responded to the sensitivity of the odor sensors. Thus, SPME was introduced to identify volatile components. SPME can enrich trace components and has the advantages of rapid environmental protection and convenience. It is commonly used for the detection of volatile components.

**Figure 4:**
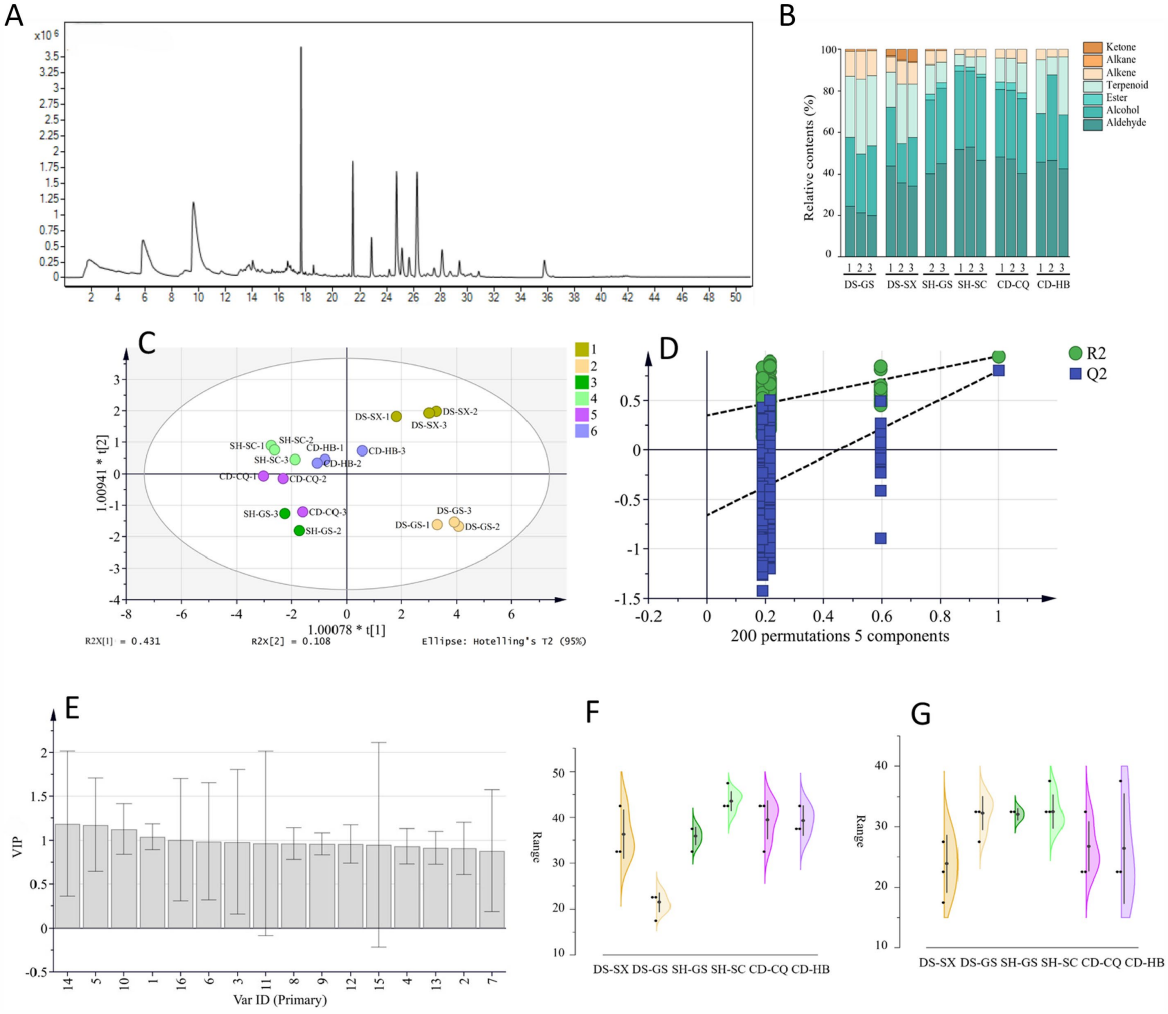
OPLS-DA analysis of SPME-GC–MS. **(A)** Total ion chromatogram (DS-GS-1). **(B)** Stacked histograms. **(C)** OPLS-DA analysis of geographical origins. **(D)** 200 permutation test of OPLS-DA. **(E)** VIP value of OPLS-DA. **(F)** Relative content of hexanal. **(G)** Relative content of 1-hexanol.

Referring to the above experimental method, some samples were selected for detection, and the total ion chromatogram of *Codonopsis Radix* is shown in Figure 4A, taking DS-GS-1 as an example. After that, we used the NIST14 database for search and comparison, counted volatile compounds with a match greater than 85%, and calculated relative contents. Seven types of compounds were matched, namely, aldehyde, alcohol, ester, terpenoid, alkene, alkane, and ketone. Stacked histograms (Figure 4B) showed that aldehyde and alcohol accounted for a higher percentage, and their main components were hexanal and 1-hexanol, indicating that hexanal and 1-hexanol were the main volatile components of *Codonopsis Radix*, which was consistent with related studies.

#### 3.3.2 OPLS-DA analysis

SIMCA 14.1 was used to analyze the relative content of volatile compounds in different *Codonopsis Radix* by OPLS-DA (Figures 4C,D). *R^2^x* was 0.860, *R^2^y* was 0.801, and *Q^2^* was 0.569, and the permutation test was effective. The results of VIP values are shown in Figure 4E. It can be seen that hexanal (peak 1) and 1-hexanol (peak 5) were statistically significant for distinguishing *Codonopsis Radix* from different origins, and the VIP values were 1.042 and 1.176, respectively. Therefore, hexanal and 1-hexanol could be used to identify the origins of *Codonopsis Radix*, and their relative contents in different *Codonopsis Radix* samples are shown in Figures 4F,G.

### 3.4 SPME-GC–MS and E-nose correlation analysis

**Figure 5:**
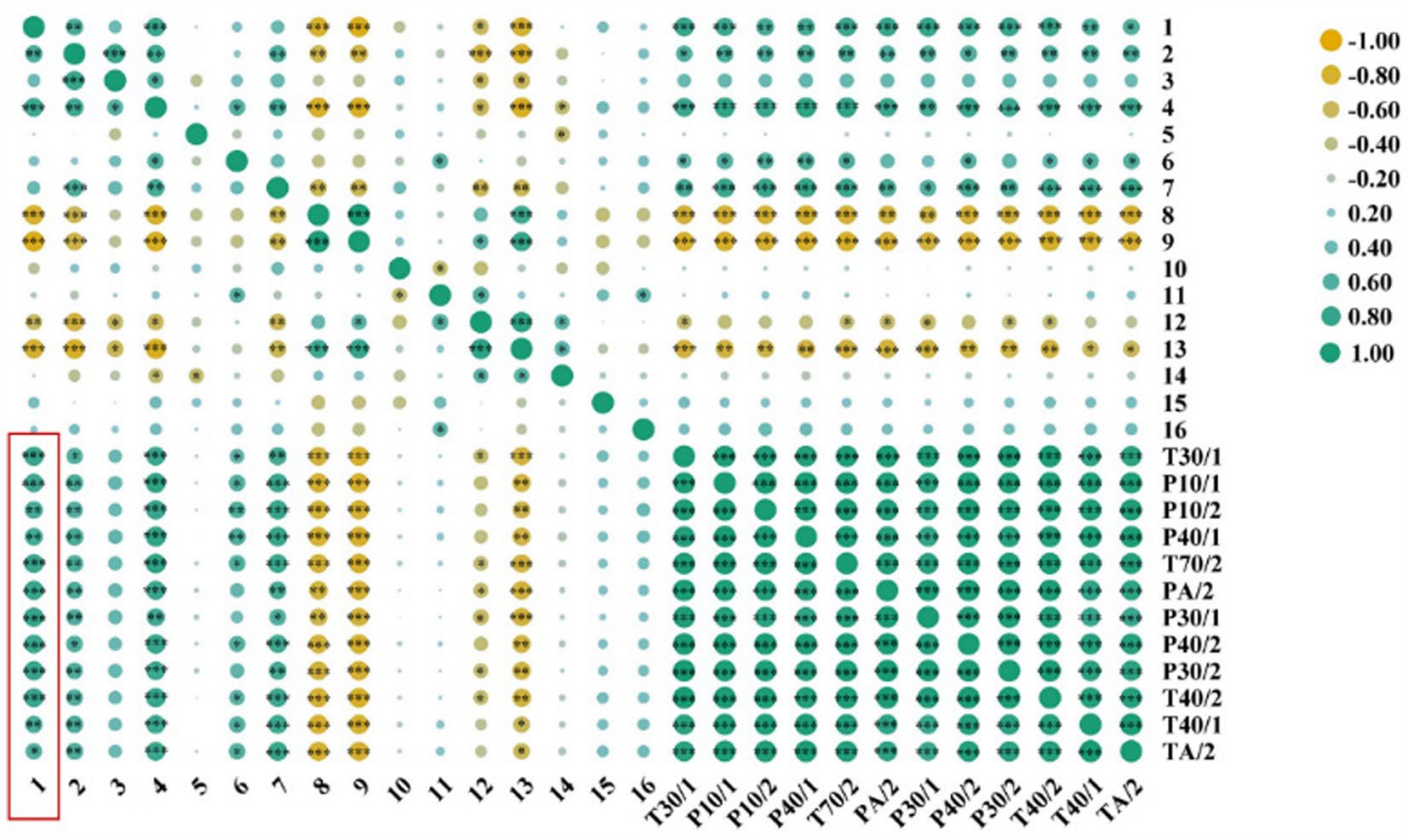
Correlation heat map (* < 0.05, ** < 0.01, and *** < 0.001).

The Pearson correlation analysis was performed between the 16 compounds matched by SPME-GC–MS and the 12 sensors with large E-nose response values. Then, the correlation heat map was plotted (Figure 5). Green indicated a positive correlation, and yellow indicated a negative correlation, and the larger the spot, the more significant the correlation. From the heat map, we could see that the correlation between hexanal (peak 1) and the 12 sensors with larger E-nose response values was tight, but the correlation between 1-hexanol (peak 5) and them was weak. Taken together, these results suggested that hexanal might be the main component of the special aroma of *Codonopsis Radix* and was the main volatile component of different *Codonopsis Radix* samples, as well as the main volatile component of E-nose sensors that produce different responses. In addition, studies have shown that the special smell of hexanal is related to the aroma of tea (14), the quality of tomatoes (15), the flavor of biscuits (16), etc., and is a volatile compound that deserves close attention. Then, does hexanal correlate with metabolic pathways of common efficacious components with health effects in plants, such as Codonopsis polysaccharides and Lobetyolin? This will be a key scientific question for subsequent in-depth research.

## 4 Discussion

The above results indicated that odor information could replace chemical components to identify *Codonopsis Radix’s* geographical origins, and the suggested classification algorithms are MultiClassClassifier and Logistic. In this study, the accuracy of origins classification for 77 batches of *Codonopsis Radix* using these two classification algorithms was 100%. In addition, we clarified that the differential volatile compounds of *Codonopsis Radix* are dominated by hexanal.

E-nose can simulate the human sense of smell with high sensitivity and objectivity and is able to capture subtle odor differences, thus helping to identify information from samples. However, constructing a sample origin identification model based on odor information requires a large number of samples as data support, and in order to continue to enrich the database behind the model, more *Codonopsis Radix* samples from other origins will be included subsequently to further improve the accuracy of model’s discriminatory and predictive capabilities. In addition, the key constituent structure of the E-nose is the gas sensor array, and through correlation analysis, important sensors used for origin identification of *Codonopsis Radix* can be screened out. This study laid the foundation for the subsequent development of portable E-noses equipped with a small number of sensors for rapid and quasi-testing (17).

*Codonopsis Radix*, as the homology of medicine and food, has gradually entered the kitchen from pharmacies (18). With the advancement of science and technology and increasing demand for a healthy diet, *Codonopsis Radix* is expected to be further developed into a variety of nutritious and functional food products, such as *Codonopsis pilosula* tea, *Codonopsis pilosula* pastries, and *Codonopsis pilosula* oral solution, to satisfy health needs of different consumer groups. The quality of *Codonopsis Radix* is the key to research and development. Subsequently, this study will be used as the basis for an in-depth study on the association between volatile components and efficacious components with health effects, which will provide a way of thinking for the quality evaluation of *Codonopsis Radix*. In addition, the results of this research can also be generalized to the research on the same types of agricultural products or other Chinese herbal medicines.

## Data Availability

The original contributions presented in the study are included in the article/supplementary material; further inquiries can be directed to the corresponding authors.
